# Pilot Study of a Surgical-Oncology Geriatric Co-Management Program

**DOI:** 10.1093/geroni/igaa057.481

**Published:** 2020-12-16

**Authors:** Megha Patel, Stephanie Chow, Lizette Munoz

**Affiliations:** 1 Icahn School of Medicine at Mount Sinai Hospital, New York, New York, United States; 2 Icahn School of Medicine at Mount Sinai, New York, New York, United States; 3 Mount Sinai Hospital, New York, New York, United States

## Abstract

Purpose: This study aims to evaluate clinical outcomes of a pilot co-management model for patients 65 years and older that were referred by their surgical oncologist for a comprehensive geriatric assessment prior to surgery. Methods: A retrospective chart review was conducted for 9 patients. Patients’ pre-operative Charlson Comorbidity Index (CCI) and frailty index were measured. Additional measures included advanced care planning (ACP) documentation and whether patients transferred primary care. Post-operative courses and complications were followed, including length of stay (LOS) and discharge outcomes. Results: A total of 9 patient charts were reviewed. The average age was 79 years. The average CCI and frailty indices were 9 and 4, respectively. Every patient had ACP during the initial assessment. Five patients had multiple outpatient geriatrician visits. Of the 9 referrals, 7 proceeded with surgery. LOS ranged from 6 – 22 days, with a median and average of 8 and 11 days, respectively. Of those undergoing surgery, 4 had an inpatient geriatrics consult. Complications included 1 mortality, 2 aborted cases and 4 with other complications. Four patients were discharged to previous living situations and 2 to SAR. Two patients had one ED/UC visit and 2 had multiple readmissions. No patients transferred their primary care. Conclusion: This is a small pilot showing a promising collaboration between geriatrics and surgical oncology. It outlines a supportive framework for initial and peri-operative geriatric assessments with favorable experiences for both providers. More studies are necessary to make clinical associations with this co-management model.

